# Angiotensin II during Experimentally Simulated Central Hypovolemia

**DOI:** 10.3389/fcvm.2016.00006

**Published:** 2016-03-03

**Authors:** Theo Walther Jensen, Niels Vidiendal Olsen

**Affiliations:** ^1^Department of Neuroscience and Pharmacology, The Health Faculty, University of Copenhagen, Copenhagen, Denmark; ^2^Department of Neuroanaesthesia, The Neuroscience Centre, University Hospital of Copenhagen (Rigshospitalet), Copenhagen, Denmark

**Keywords:** angiotensin II, central hypovolemia, angiotensin II receptor subtype 2, losartan, organ protection

## Abstract

Central hypovolemia, defined as diminished blood volume in the heart and pulmonary vascular bed, is still an unresolved problem from a therapeutic point of view. The development of pharmaceutical agents targeted at specific angiotensin II receptors, such as the non-peptidergic AT_2_-receptor agonist compound 21, is yielding many opportunities to uncover more knowledge about angiotensin II receptor profiles and possible therapeutic use. Cardiovascular, anti-inflammatory, and neuroprotective therapeutic use of compound 21 have been suggested. However, there has not yet been a focus on the use of these agents in a hypovolemic setting. We argue that the latest debates on the effect of angiotensin II during hypovolemia might guide for future studies, investigating the effect of such agents during experimentally simulated central hypovolemia. The purpose of this review is to examine the role of angiotensin II during episodes of central hypovolemia. To examine this, we reviewed results from studies with three experimental models of simulated hypovolemia: head up tilt table test, lower body negative pressure, and hemorrhage of animals. A systemic literature search was made with the use of PubMed/MEDLINE for studies that measured variables of the renin–angiotensin system or its effect during simulated hypovolemia. Twelve articles, using one of the three models, were included and showed a possible organ-protective effect and an effect on the sympathetic system of angiotensin II during hypovolemia. The results support the possible organ-protective vasodilatory role for the AT_2_-receptor during hypovolemia on both the kidney and the splanchnic tissue.

## Introduction

Central hypovolemia, defined as diminished blood volume in the heart and pulmonary vasculary bed (1), is a potentially life-threatening condition that left untreated leads to hemorrhagic shock (2). However, the therapeutic strategy for central hypovolemia is debated and still unresolved (3, 4). Expanding our knowledge about the hormones that act to stabilize the hemodynamic conditions during hypovolemia is essential to proposing new therapeutic strategies.

During World War II, an increase in the interest in hypovolemic shock gave rise to a large body of research on the hemodynamic effects of central hypovolemia (5–8). This research shed light on many of the now well-known hemodynamic effects of central hypovolemia and developed the broadly used experimental models for simulating central hypovolemia. There are three experimental models generally accepted as valid: head up tilt table, hemorrhage, and lower body negative pressure (1, 9). Head up tilt is an experimental model in which subjects, in the supine position, are raised to a head up position of various degrees, thereby allowing the larger part of the blood volume to move toward the lower extremities inducing an experimentally simulated central hypovolemia (1, 10). Lower body negative pressure consists of a design that induces negative pressure surrounding the lower extremities thereby driving the blood from the thoracic circulation to the lower extremities. The third experimental model is the hemorrhage in animals to a point of registered central hypovolemia. Trials using these experimental models investigated the endocrine effects of central hypovolemia, uncovering the now well-established rise in epinephrine, aldosterone, and angiotensin II during the late phase of central hypovolemia (1).

Angiotensin II, the end product of the renin–angiotensin system (RAS), is a hormone involved in maintaining blood pressure. Under normal circumstances, this system works by increasing fluid intake through dipsogenic effect mediated by the subfornical organ and by retaining sodium and water by the adrenal release of aldosterone along with a direct effect on the proximal tubule (11–14).

Angiontensin II increases the activity of the sodium–hydrogen antiporter in the proximal tubule and causes a vasoconstriction of the efferent and afferent arteriole leading to a shift of the Starling forces. Both of these effects favor water and sodium reabsorption (12, 13, 15). During central hypervolemia, angiotensin II acts as a vasopressor by inducing peripheral vasoconstriction (14, 15). Angiotensin II acts through four-subtype receptors label AT_1–4_.

In the early 90s, a growing number of pharmaceutical agents regulating the synthesis or blocking specific receptors of angiotensin II where developed. By the use of these agents during experimentally simulated central hypovolemia, it was possible to isolate different aspects of angiotensin II’s effects and locate them to specific subtype receptors. The use of an antagonist of the angiotensin II receptor subtype 1 (AT_1_-receptor) during simulated central hypovolemia resulted in a drop in mean arterial pressure, total peripheral resistance, and systemic vascular resistance. This lead to the conclusion that the G-coupled AT_1_-receptor localized in cardiac, vascular, pulmonary, renal, and adrenal tissue AT_1_-receptor is the main mediator of the vasoconstrictor effect of angiotensin II during hypovolemia (15–25). The role of the G-coupled AT_2_-receptor during hypovolemia is still not certain (15). Still no apparent studies have been carried out using a selective AT_2_-receptor agonist in experimentally simulated central hypovolemia. The non-peptidergic AT_2_-receptor agonist compound 21 is yielding many opportunities to uncover more knowledge about angiotensin II receptor profiles and possible therapeutic use.

Angiotensin II can be cleaved to Angiotensin (1–7) by the normally sparse enzyme Angiotensin Conversing Enzyme 2 (26). Angiotensin (1–7) is postulated to counteract the pressor effect of Angiotensin II and via the G-coupled Ang(1–7) receptor (26–28).

A number of studies have reported that losartan, the most common AT_1_-receptor antagonist, accentuated the hypotensive response to orthostatic stress compared with other AT_1_-receptor antagonists and angiotensin-converting enzyme (ACE) inhibitors (18, 24, 25). Such an effect may indicate that losartan in itself have an effect beyond AT_1_-receptor antagonism. These results have since given rise to several different hypotheses and explanations to why losartan and other AT_1_-receptor antagonists have an accentuated orthostatic hypotensive effect compared with other general angiotensin receptor antagonists and ACE-inhibitors (18, 22, 24, 25, 29). The studies of the effect of losartan have revived older debates on the interaction of angiotensin II with the sympathetic system. It is undisputed that angiotensin II has a blunting effect on the baroreflex, but whether this is mediated either centrally or peripherally is still disputed (30–34).

The purpose of this review is to examine the role of angiotensin II during episodes of central hypovolemia.

## Methods

**Figure 1:**
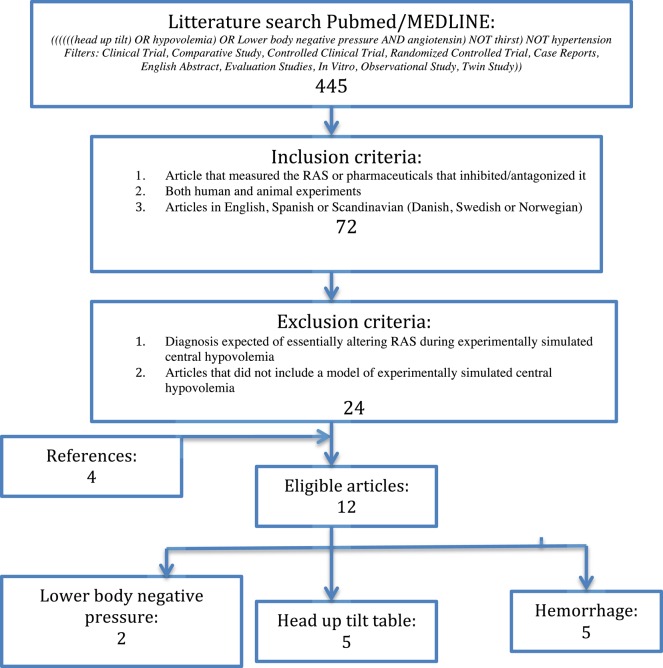
**Flowchart of study selection**.

The search was executed using the search terms*:* “*((((((head up tilt) OR hypovolemia) OR Lower body negative pressure AND angiotensin) NOT thirst) NOT hypertension Filters: Clinical Trial, Comparative Study, Controlled Clinical Trial, Randomized Controlled Trial, Case Reports, English Abstract, Evaluation Studies, In Vitro, Observational Study, Twin Study))*” in pubmed/MEDLINE. The latest search was performed in October 2015. This approach provided a result of 445 articles. After applying the following inclusion criteria: studies using AT_1_-receptor antagonist; both human and animal experiments; articles in English, Spanish, or Scandinavian (Danish, Swedish, or Norwegian), 72 remained. Exclusion criteria were the following: diagnosis expected of essentially altering RAS during experimentally simulated central hypovolemia (e.g., hypertension); articles that did not include a model of experimentally simulated central hypovolemia. The latter also applied with a set of cut-offs, as defined at the end of this section. The search gave eight eligible articles. Further four articles were included by reference lists. This gave a total of 12 eligible articles. Of the eligible articles, five were head up tilt table, five studies were hemorrhage of animals, and two were lower body negative pressure, as shown in Figure 1. There was a relatively even distribution between the three different designs. There was a predominance of studies with animal subjects but there was no noteworthy difference in the number of subjects (see Table 1).

**Table 1:** **List of eligible studies**.

Author	Result	Model	*N*	Subject	Use of antagonist or inhibitor
Aneman et al. (35)	During hypovolemia, AT1RA animals had improved portal venous blood flow and jejunal mucosal perfusion maintenance and better survival rate	Hemorrhage	20	Pigs – *landrace*	AT1RA
Bedette et al. (22)	Lorsartan have the same AT1RA. AT1RA’s kan cross the blood–brain barrier and can have its effect on pre-motor neurons	Head up tilt table	10–14	Rats	AT1RA
de Moura et al. (18)	The marked orthostatic cardiovascular response of losartan may be due, in part, to an interaction of this antagonist with Ang-(1–7) receptors	Head up tilt table	24	Rats – *male Wistar*	AT1RA and ACE-inhibitor
Franke et al. (36)	During the LBNP, GFR decreased significantly during cardiopulmonary stress testing in the subjects taking the placebo and remained unchanged in those under treatment with AT1RA	Lower body negative pressure	18	Human	AT1RA
Hashimoto et al. (24)	During hypovolemia losartan result in prolonged orthostatic hypotensive effect compared to other AT1RA	Head up tilt table		Rats	AT1RA
Hatton et al. (19)	Angiotensin II have an effect on the sympathetic system mediated by the AT_1_-recpetor. The site of action I peripherally	Lower body negative pressure	–	Cats	AT1RA and ACE-inhibitor
Johansson et al. (23)	Hypovolemia was seen to inhibit alkaline secretion and that angiotensin II potentiated that inhibition. Furthermore, it is argued in favor of a peripheral site of action of angiotensin II on the sympathetic nervous system	Hemorrhage	18	Rats – * Sprague-Dawley*	AT1RA and ACE-inhibitor
Laesser et al. (37)	Jeujenal perfusion is accentuated by AT1RA and activation of intestinal AT_2_-receptors may play a significant role in improving the outcome of severe hypovolemia	Hemorrhage	53	Pigs – *Landrace*	AT1RA and AT2RA
Ohlstein et al. (25)	Losartan have an effect beyond AT_1_-receptor antagonism. With accentuated orthostatic intolerance	Head up tilt table	22	Rats	AT1RA and ACE-inhibitors
Pancera et al. (16)	Losartan has inhibitory effect on the sympathetic activity and maintained vagal activity	Head up tilt table	18	Human	AT1RA
Ryckwaert et al. (21)	Selective AT_1_-receptor blockade was associated with a vasodilatation and a preservation of CO and SV. It is speculated that the dilation is due to overstimulation of the AT_2_-receptor	Hemorrhage	36	Piglets – *farm bred*	AT1RA and AT2RA
Ryckwaert and Colson (20)	The results suggest that AT_2_-receptor had only small if any contribution to a systemic vasodilatation observed during AT_1_-receptor blockade	Hemorrhage	18	Piglets – *farm bred*	AT1RA and AT2RA

*AT1RA, AT_1_-receptor antagonist and AT2RA, AT_2_-receptor antagonist*.

## Cut-Offs and Comparisons of Models

The following cut-offs are made to insure that the response to the hypovolemia models stimuli elicited a hormonal response rather than a purely baroreceptor-mediated compensation. The minimum amount of degrees raised in head up tilt table is 30, since that seems to be the lowest tilt where non-baroreceptor response has been observed (1, 32). The amount of negative pressure in lower body negative pressure models needed to sufficiently simulate central hypovolemia is disputed (38). A moderately conservative cut-off is that above −40 mmHg the organism does not treat the design as central hypovolemia (39–42). Others ­suggest −50 mmHg (43). This article will use the −40 mmHg cut-off as accepted induced pressure.

Below 20% hemorrhage of total blood volume in some species, the carotid sinus maintains mean arterial pressure and central hypovolemia will not be observed (44, 45). The temporal dimension was also considered in all three models. Although there was no clear temporal cut-off, no studies were included that did not have reasonable temporal dimensions.

Two recent studies have compared the three models and have found similar results regarding conversion of degrees in head up tilt table, mmHg negative pressure, and percentage of total blood volume (40, 43). The cut-off values chosen for each of the three models of simulated central hypovolemia is similar when converted with the conversions of these two studies.

Head up tilt table tends to produce a larger fluctuation in mean arterial pressure, which might originate from an activation of vestibular control on the cardiovascular system, whereas lower body negative pressure increases heart rate relatively more. There tend to be a higher vascular resistance change in the lower, compared to the upper, body in head up tilt, compared to lower body negative pressure, which produce an equal change in lower and upper body (46).

## Results

Explaining the accentuated orthostatic response of losartan has lead to two general areas of interest for analysis of angiotensin II’s effect during episodes of experimentally simulated central hypovolemia. One argued that, when the AT_1_-receptor is blocked, the rise in angiotensin II implies that there will be a corresponding increase in the stimulation of other angiotensin II receptors. Most relevant in this context is the AT_2_-receptor and Ang(1–7) (15, 20–22). The other argues that losartan seems to be blunting a sympathetic effect of angiotensin II (18, 22, 24, 25).

## Organ-Specific Protective Function of AT_2_-Receptor

Initially, the hypothesis was that the overstimulation of the ­AT_2_-receptor resulted in a systemic vasodilation (21, 47). Ryckwaert et al. found that, when an AT_1_-receptor antagonist was used during 20 and 40% total blood volume hemorrhage in pigs (*N* = 18), there was a significant fall in vascular resistance compared with ACE-inhibitors (21). Further selective AT_1_-receptor blockade was associated with a vasodilatation. This lead to the initial hypothesis, which seemed likely since several studies have observed that the AT_2_-receptor has a vasodilatory effect in some tissues (15, 38, 48–50). However, other studies observed that the vasodilatory effect of the AT_2_-receptor was not dominant during hypovolemia (51). Ryckwaert et al. tested the hypothesis in a later study using both AT_1_-receptor antagonists, AT_1_-receptor alone, and together with AT_2_-receptor antagonists during experimentally simulated central hypovolemia. The result did not show a significant systemic difference between the two groups leading to the conclusion that there is no systemic effect of overstimulation of the AT_2_-receptor during hypovolemia (20).

Even though there is no evidence of a systemic role of the ­AT_2_-receptor during central hypovolemia, there is some evidence supporting an organ-specific vasodilatory role for the AT_2_-receptor. Examining the kidney function during experimentally simulated central hypovolemia, it is observed that AT_1_-receptor antagonists help maintain the GFR despite a reduction in RPF and RBF compared with controls (29). This suggests a role of the angiotensin II that is not mediated by the AT_1_-receptor. The relatively high expression of the AT_2_-receptor in the kidney, intestines, and other tissues, together with findings that the AT_2_-receptor antagonist increases pressure and decreases nitric oxide concentration is in favor of an organ-specific role (15, 47, 52).

In a study in which landrace pigs (*N* = 53) underwent a 30% total blood volume hemorrhage, it was reported that survival rates were higher in the group, which were only given AT_1_-receptor antagonist, compared with the group given both AT_1_-receptor and AT_2_-receptor antagonists (37). The authors found that during AT_1_-receptor antagonism only, and hence comparative overstimulation of the AT_2_-receptor, the jejunal intraluminal nitric oxide output was significantly higher. During hypovolemia, there was a greater expression of the AT_2_-receptor in the jejunum. This indicates that AT_2_-receptor would have an organ-protective function during hypovolemia in the small intestines. A study using an AT_1_-receptor antagonist in pigs during a 40% total blood volume hemorrhage found increased jejunal perfusion and portal venous flow compared with a control group and increased survival among AT_1_-receptor antagonist-treated pigs (35).

## Possible Role for the Ang(1–7) Receptor

de Moura et al. have suggested a possible role for Ang(1–7) receptors in episodes of central hypovolemia, arguing that the augmenting effect of losartan is due to a stimulation of the Ang(1–7)-receptor. They presented evidence that the combined use of losartan with a Ang(1–7)-receptor antagonist leads to a removing of the bradycardic effect of losartan resulting in a blunting of the orthostatic response of losartan (18). Analogously Ryckwaert el al. (20) have suggested that the fall in vascular resistance observed with a AT_1_-receptor antagonist might originate from increased metabolites, such as Ang(1–7) or Ang(4), which might have vasodilatory properties (53–55).

## Sympathetic Actions of Angiotensin II during Experimentally Simulated Central Hypovolemia

The debate about sympathetic stimulation of angiotensin II is centered on the question whether angiotensin II has a central or a peripheral site of action on the sympathetic system during central hypovolemia.

That angiotensin II can have a potentiating effect on the sympathetic system is well-known (25, 56, 57). Hatton et al. was the first to suggest that angiotensin II has a sympathetic effect during hypovolemia. They observed a prolonged negative effect on arterial pressure during lower body negative pressure with ACE-inhibitors compared with an AT_1_-receptor antagonist. They further observed that there was not a reduced efferent nerve activity and, hence, suggested a peripheral site of action for angiotensin II (19).

Ohlstein et al.’s result from head up tilt table test gave the first evidence for a specific AT_1_-receptor-mediated sympathetic effect during hypovolemia. The accentuated hypotensive effect of losartan led them to suggest that the stimulation of specific receptors at the site of the primary baroreceptor synapse (25). Johansson et al. found that a moderate hemorrhage of 10% of the total blood volume induced a significant fall of nearly 50% in mucosal alkaline secretion in rats (*N* = 18). This effect could be reversed by the administration of an AT_1_-receptor antagonist and elevated by administration of exogenous angiotensin II. This suggested that angiotensin II had a prolonging effect on symphato-adrenergic inhibition of mucosal alkaline secretion acting specifically on efferent secremotor neuron in the intestines (23). Angiotensin II was administered to ACE-inhibitor-treated animals and a significant inhibitory effect was observed during direct splanchnic nerve stimulation (23). This indicates the presence of a peripheral mechanism of action (23).

Pancera et al.’s head up tilt table tests in human subjects showed that losartan inhibits the sympathetic activity and maintained vagal activity. This suggests a direct sympathetic function of the AT_1_-receptor and a vagally inhibitory function of angiotensin II acting peripherally (16).

Using head tilt table, Bedette et al. showed that conscious rats treated with losartan seem to have the same compensatory mechanism as with other AT_1_-receptor antagonists. Bedette et al. argued that AT_1_-receptor antagonists can cross the blood–brain barrier and that the reason for an accentuated orthostatic effect of losartan is due to a differentially centrally mediated action of the AT_1_-receptor antagonists (22, 58).

## Discussion

The evidence suggests an organ-protective function of angiotensin II in splanchnic tissue, most prominently the kidney and the intestines. The results of investigating angiotensin II’s interaction with the sympathetic system during experimentally simulated central hypovolemia are indicating both centrally and a peripherally mediated inhibition of the baroreflex (16, 23).

There is evidence supporting the organ-protective role of the AT_2_-receptor in studies that are not using one of the three models of hypovolemia (47). As splanchnic ischemia and acute renal failure are recognizable factors in postoperative morbidity (59), a possible protective function yield a number of possible uses for AT_2_-receptor agonists (60). One promising candidate for an AT_2_ agonist is the non-peptide “Compound 21” developed by Vicore Pharma AB (61).

Although seemingly not addressed so far, future pharmaceutical use, using one of the models for central hypovolemia might be relevant if further evidence support an organ-protective role during hypovolemia (35) and potentially opening the use of this and other AT_2_ agonist in hypovolemic therapy.

Ang(1–7) could represent an explanation for some of the vasodilatory actions seen with AT_1_-receptor antagonists. There are arguments for such an effect, including losartan-sensitive Ang(1–7) receptors, in the heart (62) and kidney (55, 63). However, the results contraindicate this finding (64) and de Moura et al.’s conclusion is seemingly that the effect is at best minor (18).

Studies have shown both peripheral and central mechanism of Angiotensin II in studies with one of the models of hypovolemia.

Bedette et al.’s argue that the effect of AT_1_ antagonist is obscured by the anesthetic blunting of the sympathetic system. They present the suggestive hypothesis that AT_1_ antagonists depending on pharmacological specificity might give differential interference with central pre-motor neurons. This cannot be ruled out since the AT_1_ antagonist is able to pass the blood–brain barrier and could lead to the altered response. However, the anesthetic effect cannot explain the physiological evidence for both centrally (65–69) and some peripheral site of action (34, 70) in studies not using one of the three models.

## Conclusion

There is evidence supporting an organ-protective function of the AT_2_. A study with one of the three accepted models of simulated hypovolemia could be used to verify this, with an AT_2_ agonist such as compound 21 (61). The verification of an ­organ-protective function could yield the use of such agents in hypovolemic therapy.

Further studies aimed at distinguishing between a ­AT_2_-receptor stimulation and a Ang(1–7) stimulation could prove useful to clarify the presence of a synergistic effect.

There is evidence supporting that the blunting of the baroreflex by angiotensin II is a combination of peripheral and central mechanism and that it is mediated by the AT_1_-receptor. Future trials investigating angiotensin II’s sympathetic actions with one of the three accepted models of simulated hypovolemia would help clarify the exact site of action and the strength of this blunting in hypovolemic patients. Such result is advantageous in determining possible interactions and side effects of future pharmaceutical agents.

## Author Contributions

TJ and NO have both contributed to designing and planning the work process. The idea for the article originated from NO. TJ conducted the literature search and first draft in December 2014. In early 2016, NO revised the data and contributed with much of the intellectual and academic input for the second draft. A new search was done in October 2015, and the final draft was approved by both authors after academic scrutiny.

## Conflict of Interest Statement

The authors declare that the research was conducted in the absence of any commercial or financial relationships that could be construed as a potential conflict of interest.
